# The Key to Longevity? Having Long-Lived Parents Is a Good Start

**DOI:** 10.1371/journal.pbio.0040119

**Published:** 2006-04-04

**Authors:** Liza Gross

Many studies show that tweaking a single gene can extend life span in the worm and other model organisms. That's nice for them, you may say, but what about humans? It stands to reason that if manipulating a key gene can increase longevity in these animals, humans may well harbor genetic variants, or alleles, that confer some protective advantage to the same end.

In a new study, Gil Atzmon, Marielisa Rincon, Nir Barzilai, and their colleagues followed this logic to look for genetic clues to longevity in a group of 214 Ashkenazi Jews who have passed or nearly reached the century mark. Since centenarians are not prone to cardiovascular disease, diabetes, and other age-related disorders, the researchers reasoned, it's likely that they possess protective genotypes that increase the likelihood of reaching a ripe old age. And if this is the case, these genotypes should occur with higher frequency in centenarians than they do in the rest of us. And, indeed, the researchers found a specific genetic profile, or genotype, that was associated with cardiovascular health, lower incidence of hypertension, greater insulin sensitivity, and longevity.

Ashkenazi Jews were recruited for the study because genetic and historical evidence suggest that the population descended from a founder group of just 30,000 or so people 500 years ago. Populations derived from a very narrow founder group tend to be more genetically homogenous than other populations, simplifying the challenge of linking a genotype to its physical manifestation (phenotype). Since longevity runs in families, the researchers could circumvent the obvious problem with finding a control group age-matched to the centenarians by recruiting children of the centenarians and then finding other Ashkenazi Jews the same age to serve as the controls.

Each participant received a physical examination and had blood drawn for genotyping and measuring levels of cardiovascular disease markers, including insulin, cholesterol, triglycerides, high-density lipoproteins (HDL, the “good” cholesterol), low-density lipoproteins (LDL, the “bad” cholesterol), and concentrations of two lipoprotein components, called apolipoproteins (APO). In a previous study, the researchers had found that centenarians' lipoproteins were larger than normal, so they also measured LDL and HDL particle size, too.

To identify genotypes that might be associated with a longevity-conducive genotype, they focused on single nucleotide polymorphisms (SNPs) in 36 genes involved in lipoprotein metabolism and other pathways linked to cardiovascular disease. This analysis revealed a polymorphism in a gene with a clear pattern of age-dependent frequency: *apolipoprotein C3 (APOC3)*. The polymorphism replaces an A (adenine) nucleotide with a C (cytosine) in the gene's promoter region, where transcription is initiated. The frequency of the *APOC3* polymorphism (CC) occurring in both copies of the gene was 25% among centenarians, 20% in their offspring, and 10% in controls.

APOC3 proteins are a major component of very low density lipoproteins (VLDL, another type of bad cholesterol) and also occur in HDL. Recent reports have linked elevated APOC3 protein levels (linked to an insulin-resistant form of the gene) to increased risk of cardiovascular disease, along with various *APOC3* polymorphisms, which did not change APOC3 levels. Given the pattern observed here and both genes' role in lipoprotein metabolism, the researchers expected that this genotype would have a protective effect and that carriers would have a favorable lipoprotein profile. And, indeed, all participants carrying the *APOC3* CC polymorphism had better triglyceride and cholesterol levels, as well as the beneficial particle size. This favorable profile corresponded to about 30% lower APOC3 serum levels.

And unlike the recently reported insulin-resistant *APOC3* genotype, this genotype corresponds to greater insulin sensitivity. Since insulin inhibits *APOC3* transcription, this may explain why APOC3 serum levels were lower in individuals carrying two copies of the allele. These individuals also had a significantly reduced prevalence of hypertension.

Altogether, the statistical associations between *APOC3* and longevity and the significant links between favorable lipoprotein-related traits and longevity strongly suggest the genotype's multifaceted contribution to cardiovascular health and longevity. Functional studies can now address whether the *APOC3* polymorphism directly influences APOC3 levels and the observed benefits or flags a nearby SNP that causes these effects. The genetic pathways driving longevity are unknown, but it seems clear that lipoprotein metabolism plays an important role—the favorable lipoprotein profiles reported here fall in line with studies of Japanese and Italian centenarians as well. By combining genotype studies of “exceptionally aged” individuals with functional studies of the identified genes, researchers can continue to tease apart the molecular agents of aging—and begin to develop strategies to ease the inevitable slide into our twilight years.

## 

**Figure:**
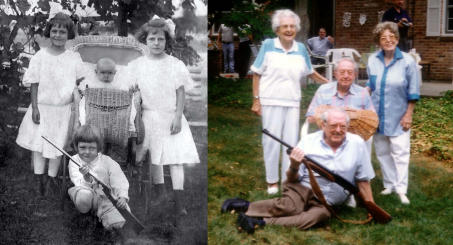
Exceptionally long-lived individuals share a common genetic feature: they posses a SNP in the promoter of a gene involved in lipid metabolism

